# Dual TBK1/IKKɛ inhibitor amlexanox attenuates the severity of hepatotoxin‐induced liver fibrosis and biliary fibrosis in mice

**DOI:** 10.1111/jcmm.14817

**Published:** 2019-12-10

**Authors:** Zixiong Zhou, Jing Qi, Jing Zhao, Chae Woong Lim, Jong‐Won Kim, Bumseok Kim

**Affiliations:** ^1^ Biosafety Research Institute and Laboratory of Pathology (BK21 Plus Program) College of Veterinary Medicine Jeonbuk National University Iksan Korea

**Keywords:** Amlexanox, hepatic stellate cells (HSCs), liver fibrosis, mice

## Abstract

Although numerous studies have suggested that canonical IκB kinases (IKK) play a key role in the progression of liver fibrosis, the role of non‐canonical IKKε and TANK‐binding kinase 1 (TBK1) on the development and progression of liver fibrosis remains unclear. To demonstrate such issue, repeated injection of CCl_4_ was used to induce hepatotoxin‐mediated chronic liver injury and biliary fibrosis was induced by 0.1% diethoxycarbonyl‐1, 4‐dihydrocollidine diet feeding for 4 weeks. Mice were orally administered with amlexanox (25, 50, and 100 mg/kg) during experimental period. Significantly increased levels of TBK1 and IKKε were observed in fibrotic livers or hepatic stellate cells (HSCs) isolated from fibrotic livers. Interestingly, amlexanox treatment significantly inhibited the phosphorylation of TBK1 and IKKε accompanied by reduced liver injury as confirmed by histopathologic analysis, decreased serum biochemical levels and fibro‐inflammatory responses. Additionally, treatment of amlexanox promoted the fibrosis resolution. In accordance with these findings, amlexanox treatment suppressed HSC activation and its related fibrogenic responses by partially inhibiting signal transducer and activator of transcription 3. Furthermore, amlexanox decreased the activation and inflammatory responses in Kupffer cells. Collectively, we found that inhibition of the TBK1 and IKKε by amlexanox is a promising therapeutic strategy to cure liver fibrosis.

## 1 INTRODUCTION

Liver fibrosis is a result of hepatic responses caused by chronic damage, in conjunction with the accumulation of extracellular matrix (ECM), low‐grade inflammation and the activation of hepatic stellate cells (HSCs).^1^, ^2^ Severe liver fibrosis can develop into liver cirrhosis, characterized by nodule formation and organ contraction.^3^, ^4^ And patients with cirrhosis are always accompanied by serious complications including portal hypertension, liver failure and hepatocellular carcinoma.^5^, ^6^ The main causes including chronic hepatitis virus B or C infection, alcohol abuse and non‐alcoholic steatohepatitis are known as leading causes to induce liver fibrosis.^3^

Among the different cell types located in liver, Kupffer cells (KCs) and HSCs are known to play a critical role in the development or regression of liver fibrosis. As the liver resident macrophages, KCs regulate inflammatory responses, which exert critical roles in progression of liver fibrosis. And KCs‐derived transforming growth factor β (TGFβ) has been suggested to induce trans‐differentiation of quiescent HSCs to activated myofibroblasts, which in turn produce other cytokines and ECM proteins. Additionally, TGFβ is one of target genes of signal transducer and activator of transcription 3 (STAT3)^7^ that promotes hepatic fibrosis through increasing TGFβ expression in mice with liver fibrosis.^8^ Also, rapid early activation of STAT3 has been observed in HSCs and fibroblasts but not in normal hepatocytes,^9^ indicating that STAT3 might be a therapeutic target for liver fibrosis. As HSCs are the key cellular resource for the production of ECM proteins and hepatic fibrosis,^10^ understanding the molecular mechanisms of HSC activation is crucial to the development of anti‐fibrotic treatments. However, the exact mechanism underlying pathogenesis of liver fibrosis is not fully understood. Also, there are still no effective drugs to treat liver fibrosis or cirrhosis.^11^

The canonical IκB kinase (IKK) complex constitutes two highly homologous catalytic subunits (IKKα and IKKβ) and one regulatory subunit (IKKγ).^12^ This kinase complex is the central regulator in nuclear factor kappa B (NF‐κB) signalling. TANK‐binding kinase 1 (TBK1) and IKKɛ, two homologous non‐canonical IκB kinases, share 27% amino acid sequence homology with IKKα/β but are not the components of the canonical IKK complex.^13^, ^14^ TBK1 and IKKɛ can phosphorylate and activate interferon regulatory factor (IRF) family of transcription factors.^15^ Activation of IRFs leads to increased production of pro‐inflammatory cytokines/chemokines and type I interferon which plays key roles in antiviral immune responses.^16^ Although many studies showed that TBK1 and IKKε are not required for NF‐κB activation,^17^, ^18^ accumulated evidence indicated that TBK1 and IKKε are involved in the activation of NF‐κB signalling pathway through phosphorylation of IκB and NF‐κB subunit p65.^19^, ^20^, ^21^ Thus, emerging evidence showed that TBK1 and IKKε have been linked with inflammatory‐related liver diseases. A recent study demonstrated that amlexanox, a dual inhibitor of TBK1 and IKKε, can reduce hepatic inflammatory reaction by inhibiting TBK1/IKKε activities, and consequently reducing obesity and insulin resistance.^22^ Also, loss of TBK1 kinase activity or IKKε protects mice from high fat diet‐induced metabolic dysfunction.^23^, ^24^

These findings led us to predict that modulation of TBK1 and IKKε and their downstream signalling pathway might affect the pathogenesis of inflammation‐related liver diseases such as liver fibrosis. This notion was further supported by recent findings, showing that inhibition of canonical IKKβ significantly attenuates hepatic inflammation and fibrogenesis in mice with non‐alcoholic fatty liver disease by reducing NF‐κB activation.^25^ However, the roles of TBK1 and IKKε in the pathogenesis of liver fibrosis are still unknown. Therefore, we aimed to clarify the precise role of non‐canonical IKKs in the development and resolution of liver fibrosis by using amlexanox.

## 2 MATERIALS AND METHODS

### 2.1 Animals

Seven‐week‐old male C57BL/6 wild‐type (WT) mice were used in this study. The mice were purchased from Taconic Farms, Inc (Samtako Bio Korea) and maintained in standard conditions (24 ± 2°C, 50 ± 5% humidity). They were fed a sterile standard chow diet and provided water ad libitum. Experimental procedures and animal management procedures were undertaken in accordance with the requirements of the Animal Care and Ethics Committees of Chonbuk National University. The animal facility of the Chonbuk National University is fully accredited by the National Association of Laboratory Animal Care.

### 2.2 Murine fibrosis induction and amlexanox treatment

Two different models of liver fibrosis were used in this study. To induce hepatotoxin‐mediated liver fibrosis, CCl_4_ (Sigma‐Aldrich; CCl_4_, 2:5 v/v in corn oil) or same volume of corn oil (Sigma‐Aldrich) were administered intraperitoneally (i.p.) at a dose of 2 mL/kg bodyweight (BW) three times per week for up to 4 weeks. To induce biliary fibrosis, mice were fed with 0.1% diethoxycarbonyl‐1, 4‐dihydrocollidine (DDC, Saeronbio Inc) for 4 weeks. Based on a previous study showing that 100 mg/kg amlexanox was no side effect to experimental mouse,^22^ different doses of amlexanox (25, 50, and 100 mg/kg) were used in our in vivo experiments. Each group of mice was orally administered with amlexanox (25, 50, and 100 mg/kg) or same volume of vehicle once a day throughout the experimental period.

After 4 weeks of DDC diet feeding, mice were fed with normal diet for the next 1, 2 and 3 weeks to establish the model of fibrosis resolution as previously described with a slight modification (the resolution [RES] group).^26^ During normal diet feeding period, mice were orally administered with amlexanox (50 mg/kg) or same volume of vehicle once a day. Prior to necropsy, animals fasted for 8 hours. Standard necropsy techniques were used, and tissues were collected and prepared for future analysis.

### 2.3 Histopathologic examination

Livers were fixed in 10% phosphate‐buffered formalin, routinely processed and then embedded in paraffin. Tissue sections (4 μm in thickness) were prepared using a microtome (HM‐340E, Thermo Fisher Scientific Inc) and placed on glass slides. Haematoxylin and eosin (H&E) staining was performed according to standard techniques.

To evaluate the severity of liver fibrosis, liver sections were stained with Sirius‐red staining (saturated aqueous solution of picric acid containing Direct Red 80; Sigma‐Aldrich). After liver sections were stained, the percentage of red‐stained collagen fibres was quantified by measuring Sirius‐red‐positive area per total liver section. Total liver section images were analysed for each animal using a light microscope (BX‐51, Olympus Corp.) and digital imaging software (analySIS TS, Olympus Corp.).

### 2.4 Biochemical measurements

To evaluate the degree of liver injury, serum levels of alanine aminotransferase (ALT) and aspartate aminotransferase (AST) were determined using AM101‐K spectrophotometric assay kits (ASAN Pharmaceutical). Absorbance values of serum biochemical were measured at wavelength of 490 nm using an EMax spectrophotometer (Molecular Devices).

### 2.5 Analysis of relative gene expression using quantitative real‐time polymerase chain reaction (qRT‐PCR)

Total RNA was isolated from cells or tissues using Easy‐Spin Total RNA extraction kit (GeneAll). Following degradation of remaining DNA using DNase I containing RNase inhibitor (Toyobo), samples were transcribed using ReverTra Ace^®^ qPCR RT Master Mix (Toyobo) according to the manufacturer's protocol. cDNA was used for real‐time PCR on a CFX96™ Real‐Time PCR Detection System (Bio‐Rad Laboratories) using SYBR Green I as a double‐strand DNA‐specific binding. After the reaction was completed, specificity was verified by melting curve analysis. Quantification was performed by comparing Ct values of each sample normalized to Ct value of glyceraldehyde‐3‐phosphate dehydrogenase. Sequences of PCR primers are listed in Table 1.

**Table 1:** Primer sequence of qRT‐PCR

Gene	Forward	Reverse
TBK1	5′‐AAGTTGATGAAGGTCAACCTGGAAG‐3′	5′‐CCTGCTGCTGATGTCCTGAAG‐3′
IKKε	5′‐GGAGTGTGTGCAGACGTATCAGG‐3′	5′‐AATGAGATGCAGGTGGTTCTGG‐3′
TNFα	5′‐GTCTACTCCCAGGTTTCTCTTCAAGG‐3′	5′‐GCAAATCGGCTGACGGTGTG‐3′
IL‐1β	5′‐CTCGCAGCAGCACATCAACA‐3′	5′‐CCACGGGAAAGACACAGGTA‐3′
TGFβ	5′‐TGAACCAAGGAGACGGAATACAGG‐3′	5′‐GCCATGAGGAGCAGGAAGGG‐3′
Col 1α1	5′‐ACAGGCGAAACCGGTGACAG‐3′	5′‐GCCAGGAGAACCAGCAGAGC‐3′
TIMP1	5′‐TCTGGCATCTGGCATCCTCTTG‐3′	5′‐AACGCTGGTATAAGGTGGTCTCG‐3′
GAPDH	5′‐ACGGCAAATTCAACGGCACAG‐3′	5′‐AGACTCCACGACATACTCAGCAC‐3′
hCol 1α1	5′‐GCTTGGTCCACTTGCTTGAAGA‐3′	5′‐GAGCATTGCCTTTGATTGCTG‐3′
hαSMA	5′‐ATAGAACATGGCATCATCACCAAC‐3′	5′‐GGGCAACACGAAGCTCATTGTA‐3′
hGAPDH	5′‐GCACCGTCAAGGCTGAGAAC‐3′	5′‐TGGTGAAGACGCCAGTGGA‐3′

Note

Abbreviations: Col 1α1, alpha‐1 type I collagen; GAPDH, glyceraldehyde‐3‐phosphate dehydrogenase; hCol 1 α1, human alpha‐1 type I collagen; hGAPDH, glyceraldehyde‐3‐phosphate dehydrogenase; hαSMA, human alpha smooth muscle actin; IKKε, IκB kinases ε; IL‐1β, interleukin 1β; IRF3, Interferon regulatory factor 3; TBK1, Tank‐binding kinase 1; TGFβ, transforming growth factor β; TIMP1, tissue inhibitors of metalloproteinase‐1; TNFα, tumor necrosis factor α.

### 2.6 Western blot analysis

Liver tissues or cells were directly homogenized on ice with an extraction buffer (T‐PER, Thermo Fisher Scientific Inc,) for 5 minutes. After centrifugation at 13 000 *g* for 15 minutes at 4°C, protein concentration in the supernatant was measured using Pierce BCA Protein Assay kit (Thermo Fisher Scientific Inc) according to the manufacturer's protocol. Equal amounts of protein were then subjected to sodium dodecyl sulphate‐polyacrylamide gel electrophoresis (SDS‐PAGE). After transferring to polyvinylidene difluoride (PVDF) membrane, blocking was carried out using 5% bovine serum albumin in Tris‐buffered saline (20 mmol L^−1^ Tris, 150 mmol L^−1^ NaCl, pH 7.4) containing 0.05% Tween‐20 at room temperature for 1 hour. The membrane was then incubated with primary antibodies diluted 1:1000 in blocking buffer at 4°C overnight. The following antibodies were used: rabbit antimouse α‐smooth muscle actin (αSMA) antibody (Abcam), rabbit antimouse TGFβ antibody (Cell Signaling), rabbit antimouse NF‐κB or phospho‐NF‐κB (pNF‐κB, Cell Signaling), rabbit antimouse STAT3 or phospho‐STAT3 (pSTAT3, Cell Signaling), rabbit antimouse pTBK1 (Cell Signaling), rabbit antimouse pIKKε (Cell Signaling), rabbit antimouse B‐cell lymphoma 2 (Bcl2, Cell Signaling), rabbit antimouse Bax (Cell Signaling) and rabbit antimouse β‐actin (Santa Cruz Biotechnology Inc). To detect antigen‐antibody complexes, peroxidase‐conjugated secondary antibodies (Santa Cruz Biotechnology Inc) were diluted 1:2000 in blocking buffer and incubated at room temperature for 1 hour. Protein bands were visualized with enhanced chemiluminescence detection system using ImageQuant™ LAS 500 (GE Healthcare Life Science). Expression levels of protein were quantified with ImageQuant™ TL software.

### 2.7 Isolation of liver cell fractions

Liver cells were fractionated into various cell populations as described by our previous studies.^27^, ^28^ Briefly, mouse livers were digested by type I collagenase (1 mL/min) perfusion. Liver cells isolated from WT mice were suspended and centrifuged at 50 g for 3 minutes. Following centrifugation, the pellet representing hepatocytes was resuspended, filtered and washed several times using DMEM (Lonza) supplemented with 10% foetal bovine serum (FBS, Thermo Fisher Scientific Inc), 100 IU/mL penicillin and 100 μg/mL streptomycin. Viability of hepatocytes was assessed using trypan blue (Sigma‐Aldrich). It was over 85%. To further isolate hepatic cells, 3‐layer discontinuous density gradient centrifugation was performed with 20%, 11.5% OptiPrep (Sigma‐Aldrich) and buffer to obtain non‐parenchymal cell fraction, and HSC fraction, respectively. Kupffer cell (KC) fractions were positively selected from the MNC fraction by magnetic cell sorting using anti‐F4/80 antibody (Miltenyi Biotec). HSC layer between 11.5% OptiPrep and buffer was carefully collected. HSC fraction was purified by negative selection of contaminating KCs by magnetic cell sorting with appropriate antibody. Cell fractions were immediately homogenized for RNA extraction or washed twice with PBS, and resuspended in RPMI‐1640 media for cell culture.

### 2.8 Lactate dehydrogenase (LDH) assay

Cell death was evaluated using a cytotoxicity detection kit (Sigma‐Aldrich) based on the measurement of activity of LDH released from the cytosol into the culture medium following the manufacturer's instruction. Absorbance of sample was measured at wavelength of 490 nm using an EMax spectrophotometer (Molecular Devices).

### 2.9 Cell culture and treatment

Hepatocytes (5.0 × 10^5^ cells/well) or KCs (1.0 × 10^6^ cells/well) isolated from WT mice were plated into 12‐well plates and then cultured at 37°C in a 5% CO_2_ incubator with DMEM or RPMI‐1640 media containing 10% FBS, 100 IU/mL penicillin and 100 μg/mL streptomycin. To mimic in vivo condition, primary hepatocytes and KCs were co‐cultured in 12‐well trans‐well plate (Sigma‐Aldrich) at a ratio 1:4 of KCs/hepatocytes. And co‐cultured cells were treated with 0.3% CCl_4_ with or without 50 μmol L^−1^ amlexanox for 24 hours.

To investigate the effect of amlexanox to inflammation, isolated KCs (5.0 × 10^5^ cells/well) were seeded in 24 wells and treated with indicated concentration of amlexanox or vehicle for 24 hours. Lipopolysaccharide (LPS, 1 μg/mL) was used to induce inflammation in KCs.

Primary HSCs (1.0 × 10^6^ cells/well) were plated onto 12‐wells plate and cultured for up to 7 days post‐isolation for cell activation. Culture media were changed every other day. Human HSC line (LX‐2, 1.0 × 10^6^ cells/well) was routinely cultured in 12‐well plate. To evaluate the roles of IKKε/TBK1 on HSCs, quiescent (culture day 1) or activated primary HSCs (culture day 7) were treated with indicated concentration of amlexanox or vehicle for 24 hours. And LX‐2 cells were treated with 10 ng/mL human recombinant TGFβ with or without amlexanox treatment for 24 hours. To activate or inhibit STAT3, cells were treated with colivelin (1 μmol L^−1^, Cayman Chemical) or SPI (10 μmol L^−1^, BioVision, Inc) with 50 μmol L^−1^ amlexanox for 24 hours.

### 2.10 Statistical analysis

All data were expressed as the mean ± standard error. Differences between multiple groups were compared using one‐way analysis of variance (ANOVA) using SAS version 9.1 (SAS Institute Inc). Duncan's multiple range test (DMRT) was used for individual comparisons. Experimental groups marked by different letters represent significant differences among groups. Differences between two groups were compared using a two‐tailed Student's *t* test. A *P* < .05 was considered statistically significant.

## 3 RESULTS

### 3.1 Increased activities of TBK1 and IKKε were found in fibrotic livers in mice

**Figure 1:**
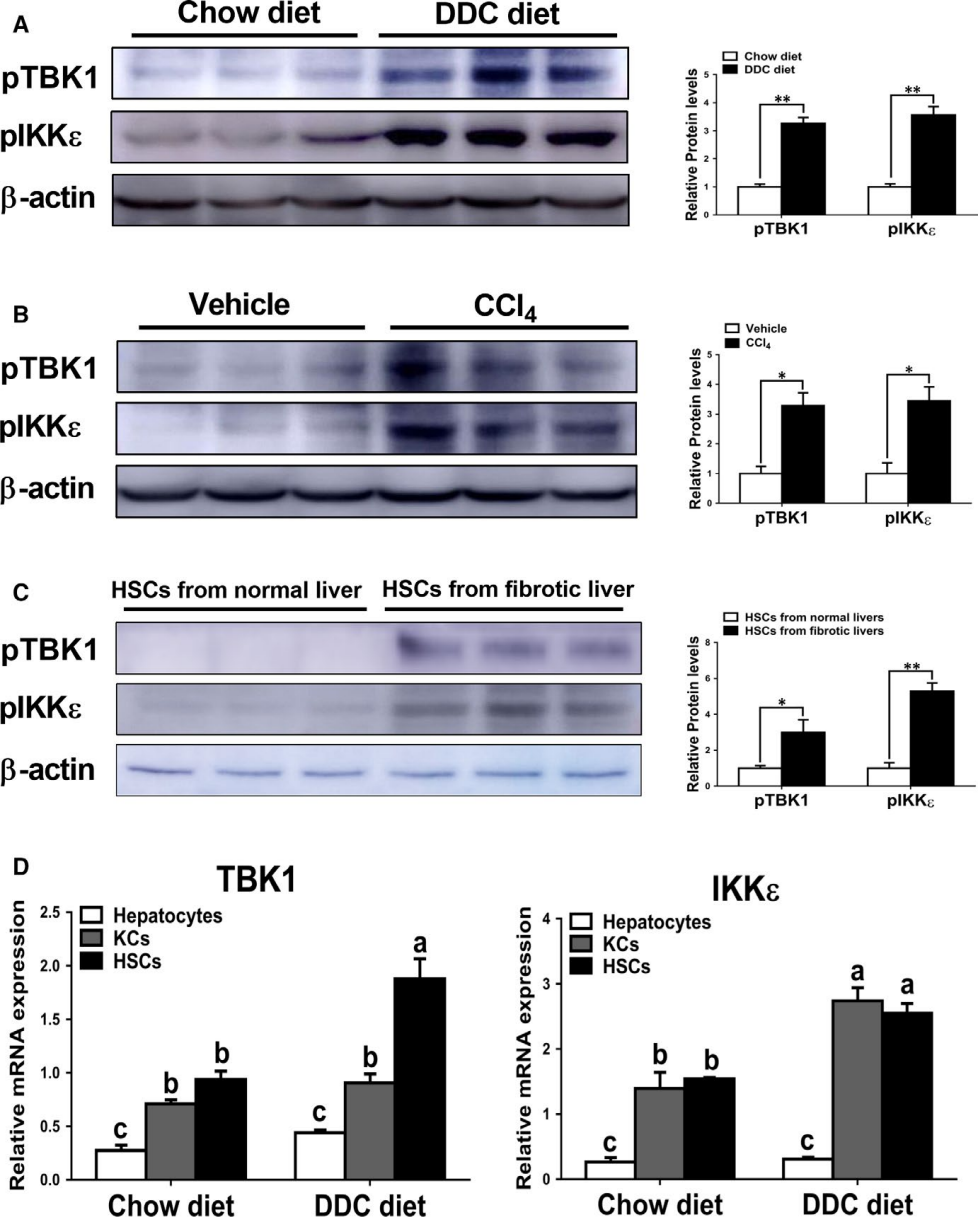
Activation of TBK1 and IKKε is closely associated with liver fibrosis in mice. A, Mice were fed with chow or DDC diet for 4 wk (n = 6 per group). Hepatic protein levels of pTBK1 and pIKKε were determined by Western blot analysis. B, Mice were i.p. injected with vehicle or CCl_4_ at a dose of 2 mL/kg BW three times per week for 4 wk (n = 6 per group). Hepatic protein levels of pTBK1 and pIKKε were assessed by Western blot analysis. C, The protein levels of pTBK1 and pIKKε in HSCs isolated from livers of mice fed with chow or DDC diet were evaluated by Western blot analysis. D, mRNA expression levels of TBK1 and IKKε in the hepatocytes, KCs and HSCs isolated from livers of mice fed with chow or DDC diet were determined by qRT‐PCR. Data are presented as means ± SEM per group. Experimental groups marked by different letters represent significant differences between groups at *P* < .05. Two‐tailed Student's *t* test, *P < .05, **P < .01

Consistent with previous finding showing increased activity of TBK1 and IKKε in the livers of mice with obesity,^22^ we also found significantly increased phosphorylation levels of TBK1 and IKKε in the livers of mice fed with DDC diet compared with those of mice fed with chow diet (Figure 1A). We observed similar patterns of phosphorylation levels of these proteins in hepatotoxin‐induced fibrotic livers (Figure 1B). To more clearly demonstrate these effects, phosphorylation levels of TBK1 and IKKε were evaluated in HSCs isolated from chow diet‐ or DDC diet‐fed mice for 4 weeks. The results showed that increased protein levels of pTBK1 and pIKKε were observed in HSCs isolated from fibrotic livers (Figure 1C). Consistently, mRNA expression levels of TBK1 and IKKε were significantly increased in HSCs isolated from fibrotic livers compared with HSCs isolated from non‐fibrotic livers (Figure 1D). Although there was no significant difference of expression levels of these genes in hepatocytes, IKKε but not TBK1 was markedly up‐regulated in KCs isolated from fibrotic livers (Figure 1D). These findings suggest that pTBK1 and pIKKε might affect the pathogenesis of liver fibrosis. These findings suggest that pTBK1 and pIKKε might affect the pathogenesis of liver fibrosis.

### 3.2 Treatment of amlexanox alleviates the severity of biliary fibrosis in mice

**Figure 2:**
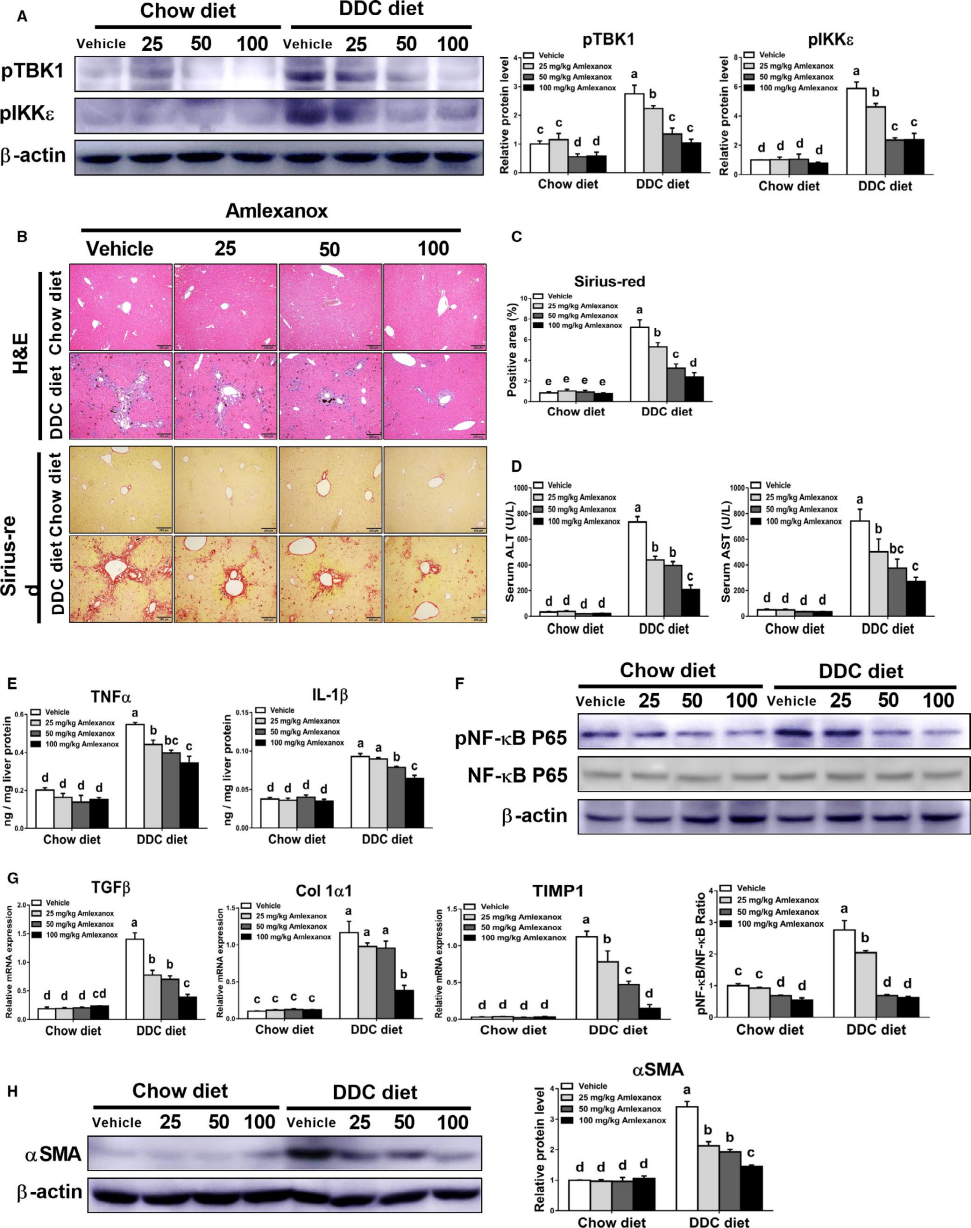
Treatment of amlexanox decreases hepatic fibrogenic responses in mice with biliary fibrosis. Mice were fed with chow diet or DDC diet for 4 wk (n = 6 per group), and chow diet‐ or DDC diet‐fed mice were orally administered with vehicle or amlexanox (25, 50, and 100 mg/kg) daily. A, The protein levels of pTBK1 and pIKKε in the livers were determined by Western blot analysis. B, Liver sections were stained with H&E or Sirius‐red to assess liver fibrosis. C, The proportion of the Sirius‐red positive area was measured. D, Serum ALT and AST levels were determined to evaluate chronic liver injury. E, The hepatic protein expression levels of TNFα and IL‐1β were determined by ELISA. F, The protein levels of pNF‐κB, NF‐κB and their ratio were determined. G, The hepatic mRNA expression levels of TGFβ, Col 1α1 and TIMP1 were evaluated by qRT‐PCR. H, The protein level of αSMA was assessed by Western blot analysis. Data are presented as means ± SEM per group. Experimental groups marked by different letters represent significant differences between groups at *P* < .05. Original magnification: ×100

To further determine the roles of TBK1 and IKKε in the pathogenesis of biliary fibrosis, chow diet‐ or DDC diet‐fed mice were orally administered with vehicle or indicated dose of amlexanox daily. The changes in bodyweight during the experimental period did not differ among the chow diet‐fed groups (Figure S1). However, reduced body weight in mice with DDC diet was recovery by treatment of amlexanox in a dose‐dependent manner. Also, DDC diet‐fed mice had decreased protein levels of pTBK1 and pIKKε in livers (Figure 2A). Based on histopathologic analysis using H&E and Sirius‐red staining, inhibition of TBK1 and IKKε by amlexanox markedly reduced biliary hyperplasia and collagen deposition in the livers of mice fed with DDC diet (Figure 2B,C). Consistent with these findings, we observed significantly lower serum levels of ALT and AST in amlexanox‐treated mice with fibrosis than those in vehicle‐treated mice with fibrosis (Figure 2D). Additionally, amlexanox‐administered mice showed dose‐dependently reduced IL‐1β and TNFα levels together with reduced NF‐κB activation in fibrotic livers. (Figure 2E,F). In line with histopathologic observation (Figure 2B,C), we found lower expression levels of pro‐fibrogenic genes such as TGFβ, alpha‐1 type I collagen (Col1α1) and tissue inhibitors of metalloproteinase‐1 (TIMP1) in fibrotic livers of mice treated with amlexanox than those of vehicle‐treated mice (Figure 2G). These results were further supported by Western blot analysis, showing that reduced protein levels of α‐smooth muscle actin (αSMA) were observed in fibrotic livers of mice treated with amlexanox (Figure 2H). These findings suggest that suppression of TBK1 and IKKε by using amlexanox can attenuate cholestasis‐induced chronic liver injury and its associated fibro‐inflammatory responses in mice through modulating NF‐κB activation.

### 3.3 Treatment of amlexanox attenuates CCl_4_‐induced liver fibrosis

**Figure 3:**
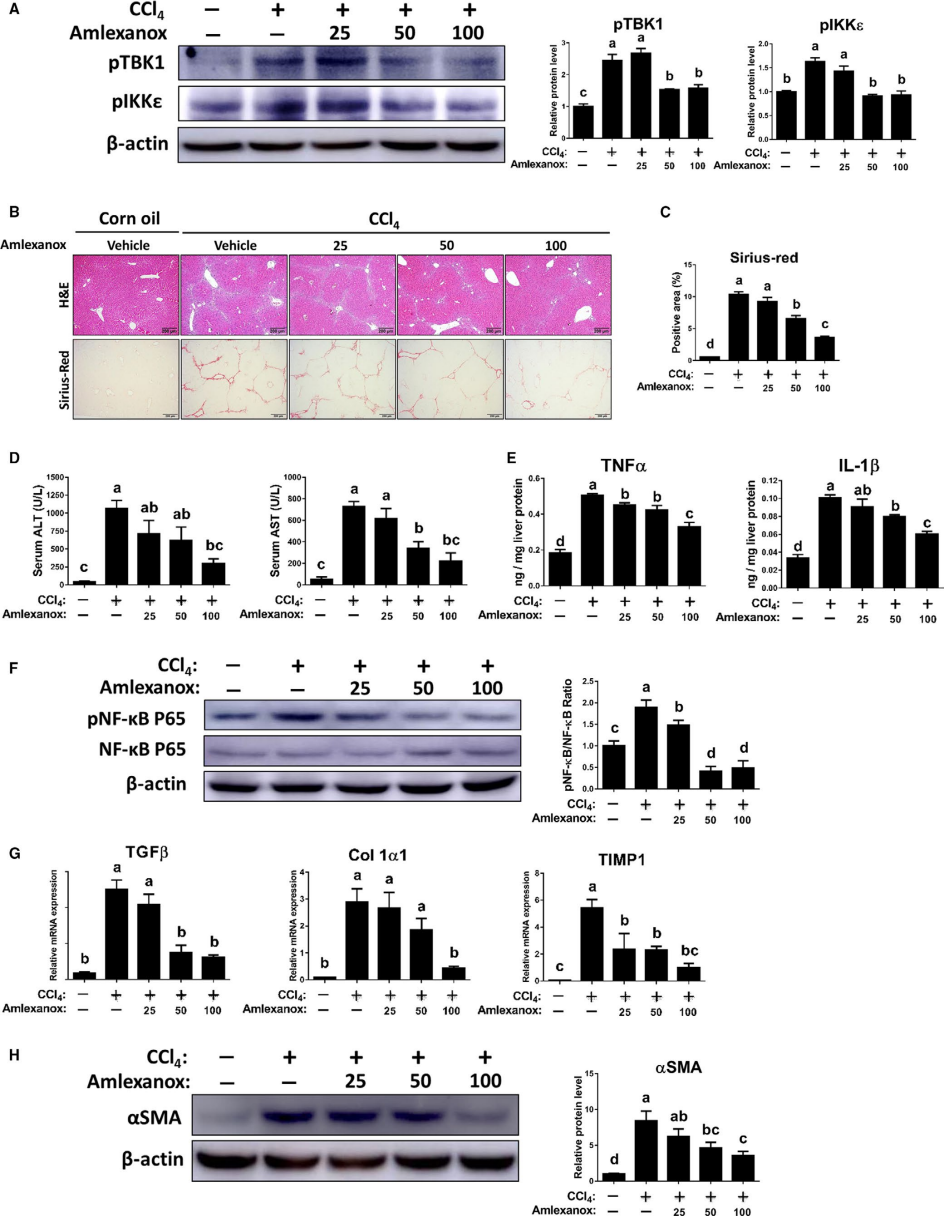
Treatment of amlexanox reduces the severity of hepatotoxin‐induced liver fibrosis. Mice were i.p. injected with vehicle or CCl_4_ at a dose of 2 mL/kg BW three times per week for 4 wk (n = 6 per group). Meanwhile, these mice were orally administered with vehicle or amlexanox (25, 50, and 100 mg/kg) daily. A, The hepatic protein levels of pTBK1 and pIKKε were determined by Western blotting. B, Liver sections were stained with H&E or Sirius‐red to evaluate hepatic fibrogenesis. C, The quantification of the Sirius‐red positive area was shown. D, Serum ALT and AST levels were determined to evaluate the degree of chronic liver injury. E, The hepatic protein expression levels of TNFα and IL‐1β were determined by ELISA. F, The protein levels of pNF‐κB, NF‐κB and their ratio were determined. G, The hepatic mRNA expression levels of TGFβ, Col1α1 and TIMP1 were determined by qRT‐PCR. H, The protein level of αSMA was determined by Western blot analysis. Data are presented as means ± SEM per group. Experimental groups marked by different letters represent significant differences between groups at *P* < .05. Original magnification: ×100

To further explore the role of TBK1 and IKKε in fibrotic responses, we next performed additional experiments using hepatotoxin‐induced liver fibrosis model. As shown in Figure 3A, amlexanox dose‐dependently decreased the protein levels of pTBK1 and pIKKε in fibrotic livers induced by CCl_4_. Similar to the results from Figure 2, inhibition of TBK1 and IKKε significantly reduced hepatic fibrogenesis and bridging fibrosis in CCl_4_‐treated mice as confirmed by histopathologic observation (Figure 3B,C). Also, amlexanox‐treated mice showed significantly lower serum levels of ALT and AST in fibrotic milieu (Figure 3D). Consistently, markedly reduced inflammation and its related NF‐κB activation were observed by amlexanox administration in the livers of mice treated with CCl_4_. (Figure 3E,F). These findings together with results from Figure 2 strongly suggest that amlexanox can exert beneficial effects in mice with liver fibrosis induced by CCl_4_.

### 3.4 Inhibition of TBK1 and IKKε promotes fibrosis resolution

**Figure 4:**
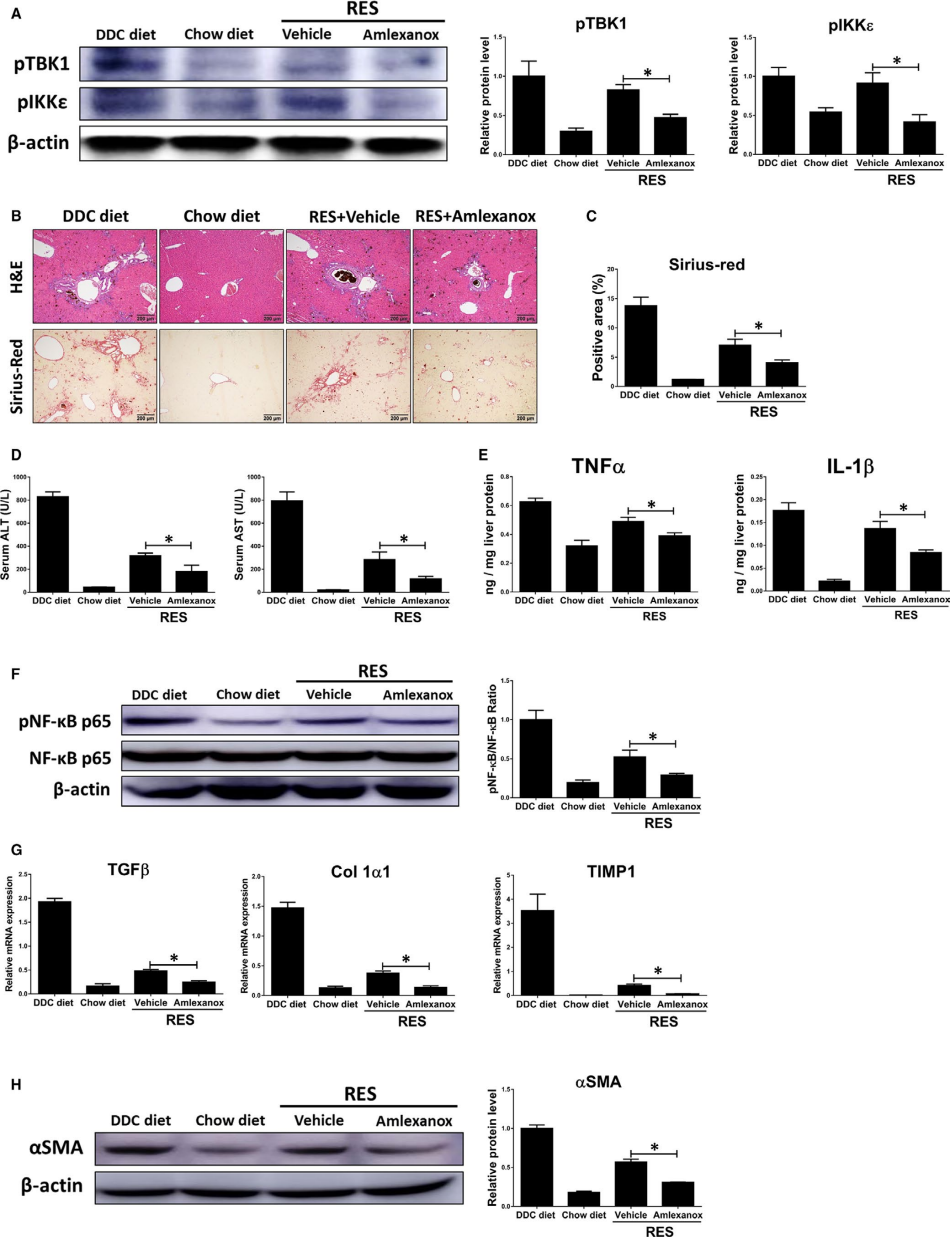
Inhibition of TBK1 and IKKε accelerates fibrosis resolution. Mice were fed with chow diet or DDC diet for 4 wk (n = 6 per group), followed by feeding a chow diet plus amlexanox (50 mg/kg) or vehicle daily for 2 wk (RES group). A, The protein levels of pTBK1 and pIKKε in the livers were determined by Western blot analysis. B, Liver sections were stained with H&E or Sirius red to assess liver fibrosis. C, The quantification of the Sirius‐red positive area was shown. D, Serum ALT and AST levels were determined to evaluate the severity of chronic liver injury. E, The hepatic protein expression levels of TNFα and IL‐1β were determined by ELISA. F, The protein levels of pNF‐κB, NF‐κB and their ratio were determined. G, The hepatic mRNA expression levels of TGFβ, Col 1α1 and TIMP1 were determined by qRT‐PCR. H, The protein level of αSMA was determined by Western blot analysis. Data are presented as means ± SEM per group. Two‐tailed Student's *t* test, *P < .05. Original magnification: ×100

As treatment of amlexanox attenuated the progression of liver fibrosis based on above results, we then investigated the roles of TBK1 and IKKε in fibrosis regression by feeding a DDC diet for 4 weeks, followed by feeding a chow diet plus amlexanox (50 mg/kg) for 1, 2 and 3 weeks (RES group; total 5, 6 and 7 weeks, respectively). Compared with 4 weeks DDC diet feeding, RES group showed markedly reduced liver fibrosis, as revealed by Sirius‐red staining. And such effects were further accelerated by treatment of amlexanox (Figure S2A). Also, we observed significantly lower expression levels of pro‐fibrogenic genes in amlexanox‐treated mice in RES group (Figure S2B). Consistently, amlexanox‐treated mice showed reduced serum biochemical and expression levels pro‐inflammatory genes during fibrosis resolution (Figure S2C,D). For more detailed analysis, we chose RES group after being shifted to a normal diet for 2 weeks. As shown in Figure 4A, significantly lower protein levels of pTBK1 and pIKKε were found in the RES treated with amlexanox. Amlexanox‐treated group showed lower liver damage and fibrogenesis in mice after 2 weeks of fibrosis resolution, as confirmed by histopathologic analysis and Sirius‐red staining (Figure 4B,C). Additionally, inhibition of TBK1 and IKKε significantly reduced serum levels of ALT and AST in mice during fibrosis resolution (Figure 4D). Significant lower inflammation was also observed in fibrotic livers of mice treated with amlexanox compared to those of mice treated with vehicle, as confirmed by reduced protein levels of pro‐inflammatory genes (Figure 4E). Consistently, administration of amlexanox resulted in significant lower protein levels of pNF‐κB by amlexanox in the livers of mice after 2 weeks of fibrosis resolution (Figure 4F). In line with histopathologic examination, RES group showed significantly lower liver fibrosis in mice treated with amlexanox, as revealed by decreased expression levels of pro‐fibrogenic genes (Figure 4G). Furthermore, we found significantly lower protein levels of αSMA in fibrotic livers of mice treated with amlexanox during fibrosis resolution (Figure 4H). These findings suggest that amlexanox can promote the resolution of liver fibrosis in mice.

### 3.5 Treatment of amlexanox significantly reduces inflammatory responses in KCs

**Figure 5:**
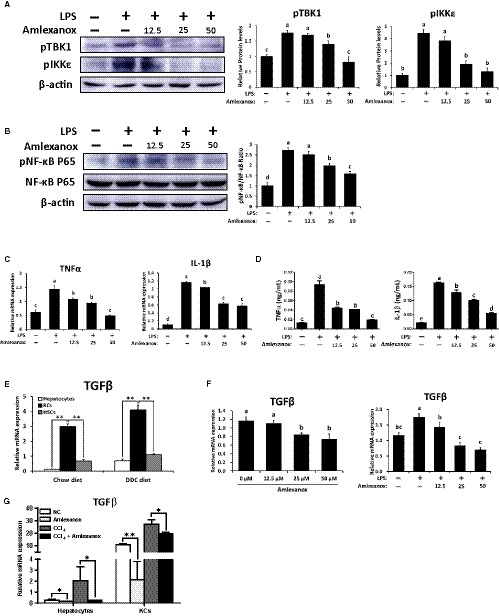
Treatment of amlexanox modulates inflammatory responses in KCs. KCs isolated from WT mice were stimulated with LPS (1 μg/mL) or PBS; meanwhile, these cells were treated with vehicle or amlexanox (12.5 25 and 50 μmol L^−1^) for 24 h (n = 8 per group). A, The protein levels of pTBK1 and pIKKε in KCs were determined by Western blot analysis. B, The protein levels of pNF‐κB, NF‐κB and their ratio were determined. C, The mRNA and D, protein expression levels of TNFα and IL‐1β in KCs were determined by qRT‐PCR. E, Hepatocytes, KCs and HSCs were isolated from chow or DDC diet‐fed mice for 4 wk, and the mRNA expression levels of TGFβ in different types of cells were determined by qRT‐PCR. F, The mRNA expression levels of TGFβ were determined in KCs with or without LPS stimulation upon amlexanox treatment. G, KCs were co‐cultured in 12‐well trans‐well plate at a ratio 1:4 of KCs/hepatocytes. And co‐cultured cells were treated with 0.3% CCl_4_ with or without 50 μmol L^−1^ amlexanox for 24 h. The mRNA expression levels of TGFβ in hepatocytes and in KCs were determined by qRT‐PCR. Data are presented as means ± SEM per group. Experimental groups marked by different letters represent significant differences between groups at *P* < .05. Two‐tailed Student's *t* test, *P < .05, **P < .01

As TBK1 and IKKε were highly expressed in KCs and HSCs (Figure 1D), we focused on KCs and HSCs. In this part, we first investigated the roles of TBK1 and IKKε on KCs in inflammatory responses. As the KCs promote the progression of liver fibrosis by producing inflammatory cytokines,^29^ the inflammatory responses were evaluated by performing in vitro study. As shown in Figure S3A,B, treatment of amlexanox significantly decreased protein levels of pTBK1 and pIKKε in KCs and its related inflammatory gene expression. To further demonstrate these effects, LPS were co‐treated with amlexanox to evaluate the role of TBK1 and IKKε on inflammation in KCs. As shown in Figure 5A, increased pTBK1 and pIKKε by LPS were markedly decreased by amlexanox in KCs. Consistently, dose‐dependently decreased protein levels of pNF‐κB were observed in LPS‐treated KCs by treatment of amlexanox (Figure 5B). Also, expression levels of TNFα and IL‐1β were decreased in LPS‐treated KCs by treatment of amlexanox (Figure 5C,D).

KC‐derived TGFβ is one of the most important cytokines involved in the pathogenesis of liver fibrosis.^29^, ^30^ Similar to these findings, TGFβ is abundantly expressed in KCs isolated from non‐fibrotic or fibrotic livers (Figure 5E). Thus, we next verified whether treatment of amlexanox affects TGFβ expression in KCs. Treatment of amlexanox significantly reduced the expression levels of TGFβ in KCs with or without LPS stimulation (Figure 5F). In addition, co‐culture experiments of hepatocytes‐KCs showed that the expression levels of TGFβ (Figure 5G) and pro‐inflammatory genes (Figure S3C) were remarkably decreased by treatment of amlexanox in CCl_4_‐mediated inflammatory milieu, and even in normal condition. Consistent with these findings, mice administered with amlexanox had significantly lower protein levels of TGFβ in fibrotic livers, as confirmed by our in vivo experiments (Figure S4). Taken together, treatment of amlexanox can suppress the production of TGFβ and pro‐inflammatory cytokines in KCs by inhibiting the activity of NF‐κB.

### 3.6 Inhibition of TBK1 and IKKε suppresses HSC activation and its related fibrogenic responses

**Figure 6:**
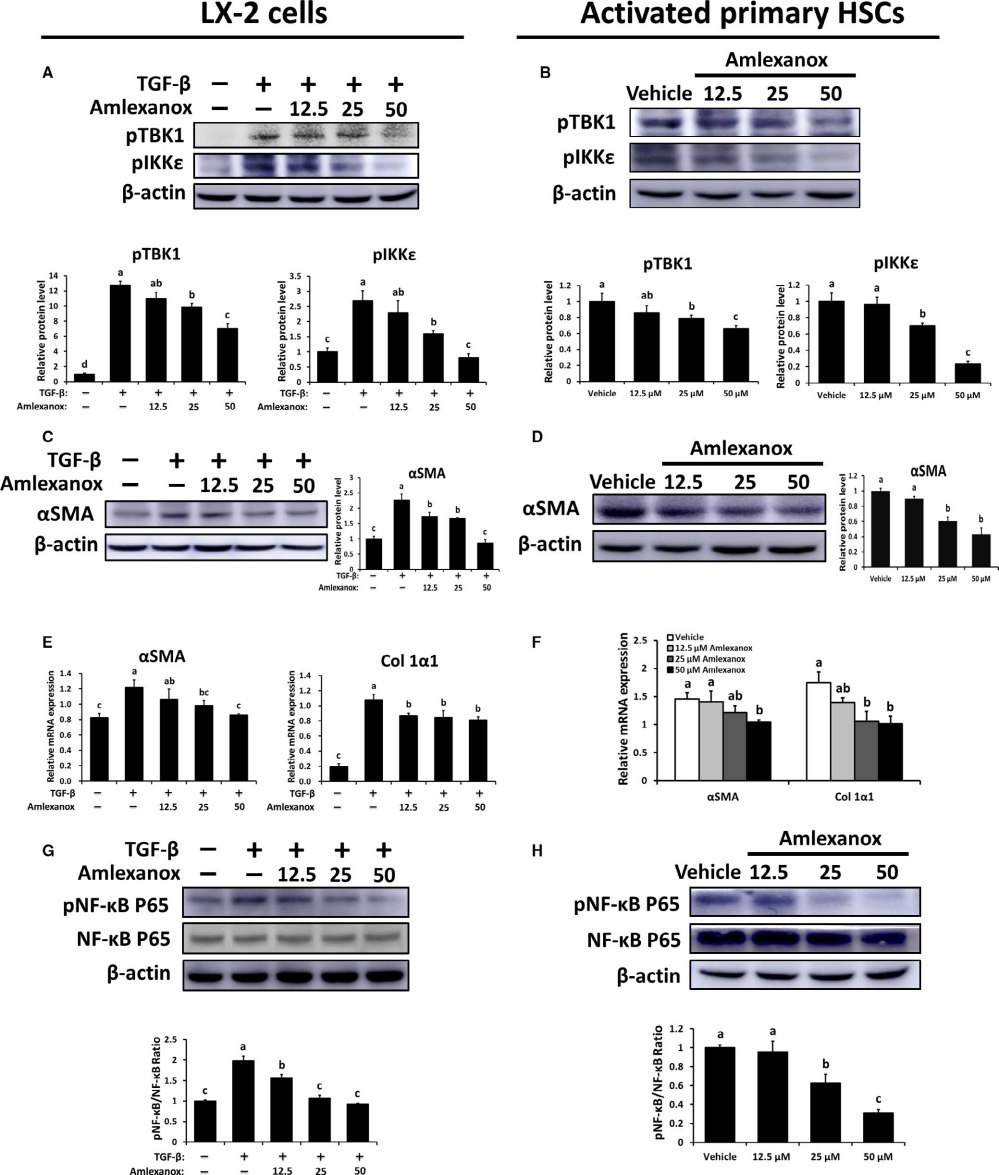
Inhibition of TBK1 and IKKε suppresses HSC activation and its related fibrogenic responses. 1 × 10^6^/well activated primary HSCs (culture day 7) and LX‐2 cells were treated with amlexanox (12.5, 25, and 50 μmol L^−1^) or vehicle for 24 h (n = 8/per group). LX‐2 cells were treated with 10 ng/mL human recombinant TGFβ for cellular activation. A and B, The protein levels of pTBK1 and pIKKε were determined in LX‐2 cells and activated primary HSCs, respectively. C and D, The protein levels of αSMA in LX‐2 cells and activated primary HSCs were assessed by Western blot analysis. E and F, The mRNA expression levels of αSMA and Col 1α1 in LX‐2 cells and activated primary HSCs were evaluated by qRT‐PCR. G and H, The protein levels of pNF‐κB, NF‐κB and their ratio in LX‐2 cells and activated primary HSCs were determined. Data are presented as means ± SEM per group. Experimental groups marked by different letters represent significant differences between groups at *P* < .05

It has been well‐known that HSCs play a critical role in the pathogenesis of liver fibrosis.^31^ Also, HSCs are one of the main cellular sources of TBK1 and IKKε (Figure 1D). Therefore, we determined the roles of TBK1 and IKKε on LX‐2 cells or primary murine HSCs by using amlexanox. As shown in Figure S5A,B, dose‐dependently decreased protein levels of pTBK1 and pIKKε also were found in non‐activated LX2 cells and HSCs upon amlexanox treatment. Also, the expression levels of pro‐fibrogenic genes were significantly decreased by treatment of amlexanox (Figure S5C,D). Next, TGFβ‐treated LX‐2 cells and activated HSCs (7 days post‐isolation) were used to determine the role of amlexanox on activated HSCs. Significantly reduced protein levels of pTBK1 and pIKKε were observed in activated LX‐2 cell and HSCs (Figure 6A,B). Additionally, inhibition of TBK1 and IKKε markedly decreased protein levels of αSMA in activated LX‐2 cells and HSCs (Figure 6C,D). Consistently, these cells showed lower expression levels of pro‐fibrogenic genes by treatment of amlexanox (Figure 6E,F). As NF‐κB is closely associated with the activation of HSCs,^32^ we next evaluated whether treatment of amlexanox affects activation of NF‐κB in HSCs and found dose‐dependently decreased protein levels of pNF‐κB in activated HSCs or LX‐2 cells upon amlexanox treatment (Figure 6G,H). These findings suggest that inhibition of TBK1 and IKKε can suppress the activation of HSCs and its related fibrogenic responses.

### 3.7 Anti‐fibrotic effects by amlexanox treatment on HSCs are partially dependent on inhibition of STAT3

**Figure 7:**
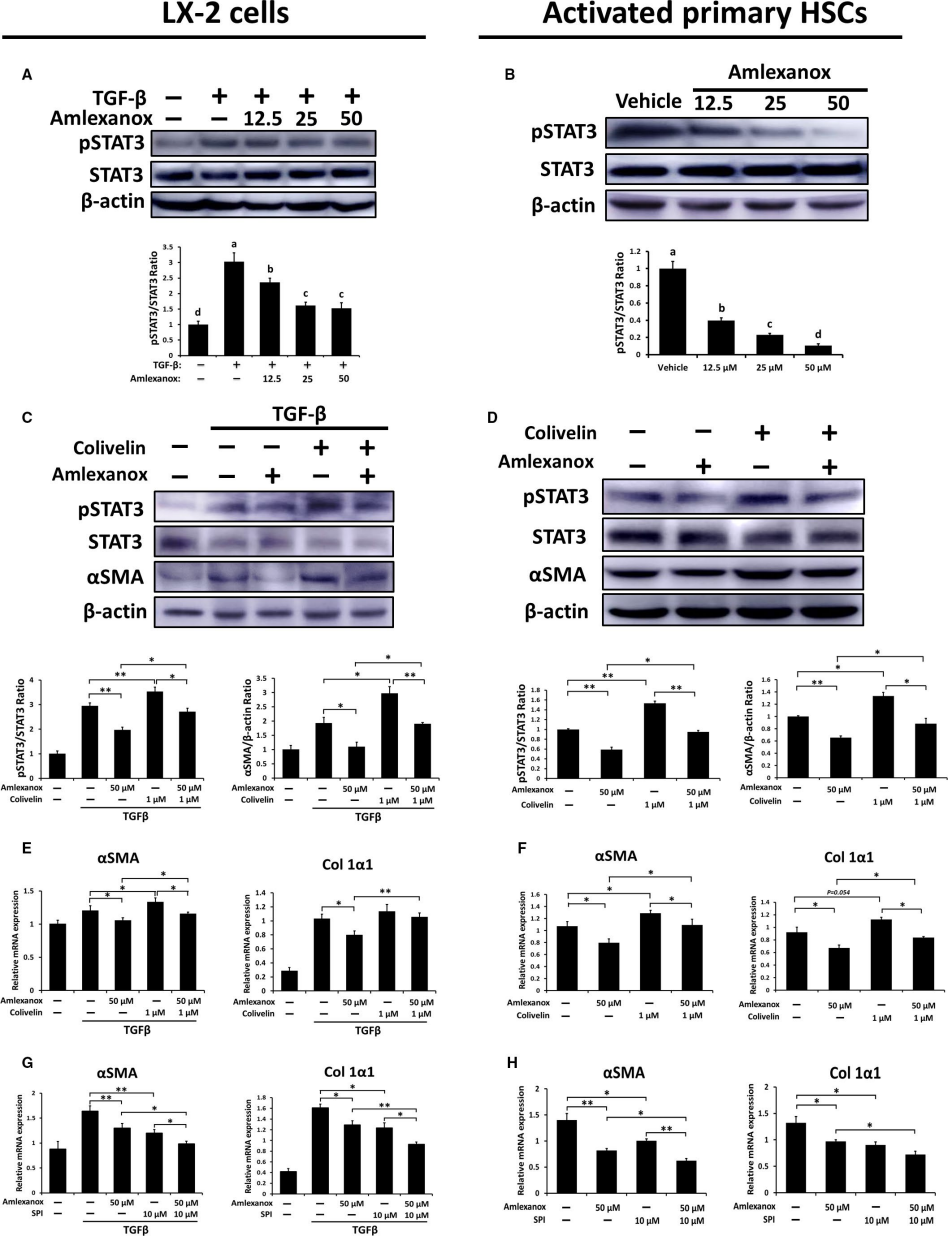
Anti‐fibrotic effects by amlexanox treatment on HSCs are partially dependent on inhibition of STAT3. A and B, The protein levels of pSTAT3, STAT3 and their ratio in LX‐2 cells and activated primary HSCs were determined by Western blot analysis. C and D, To confirm the STAT3 inhibition of amlexanox resulting in anti‐fibrotic effects, 1 × 10^6^/well activated primary HSCs and TGFβ‐treated LX2 cells were cultured with 50 μmol L^−1^ amlexanox plus 1 μmol L^−1^ colivelin for 24 h (n = 8 per group). The protein levels of pSTAT3, STAT3 and αSMA in LX‐2 cells and activated primary HSC were measured. E and F, The mRNA expression levels of αSMA and Col 1α1 in LX‐2 cells and activated primary HSCs were evaluated by qRT‐PCR. Data are presented as means ± SEM per group. G and H, To verify the STAT3 dependency of amlexanox‐mediated inhibition on HSC activation, 1 × 10^6^/well activated primary HSCs and TGFβ‐treated LX2 cells were cultured with 50 μmol L^−1^ amlexanox plus 10 μmol L^−1^ SPI (a STAT3 activation inhibitor) for 24 h (n = 8 per group), and the mRNA expression levels of αSMA and Col 1α1 in LX‐2 cells and activated primary HSCs were evaluated by qRT‐PCR. Experimental groups marked by different letters represent significant differences between groups at *P* < .05. Two‐tailed Student's *t* test, *P < .05, **P < .01

Considering that activation of STAT3 is involved in HSC activation and proliferation,^33^, ^34^ we further verified activation of STAT3 in activated LX‐2 cells and primary HSCs and found that pSTAT3 was markedly reduced when TBK1 and IKKε were inhibited (Figure 7A,B). To confirm the inhibition of STAT3 by amlexanox resulting in anti‐fibrotic effects, activated primary HSCs or TGFβ‐treated LX‐2 cells were cultured with amlexanox plus colivelin, STAT3 activator, to increase the activity of STAT3. As shown in Figure 7C,D, treatment of colivelin significantly restored reduced protein levels of pSTAT3 induced by amlexanox in activated HSCs and LX‐2 cells. Consistently, significantly decreased protein levels of αSMA by treatment of amlexanox were abolished by treatment of colivelin in activated HSCs and LX‐2 cells. Similarly, colivelin treatment reversed the decreased mRNA expression levels of pro‐fibrogenic genes by treatment of amlexanox in activated HSCs and LX2 cells (Figure 7E,F). Next, we further verified the STAT3 dependency of amlexanox‐mediated inhibition on activation of HSCs by using a STAT3 activation inhibitor (SPI). The results showed that the anti‐fibrotic effects of amlexanox were amplified by treatment of SPI in activated HSCs (Figure 7G,H). These findings indicate that amlexanox‐provided anti‐fibrotic effects on activated HSC, at least partially, are dependent on STAT3 inhibition.

## 4 DISCUSSION

Contrary to not clearly understood role of TBK1 and IKKε on liver fibrosis, the role of canonical IKKα and IKKβ were broadly understood in the progression or resolution of liver fibrosis. A previous study reported that inhibition of IKKβ attenuates hepatic fibrosis in mice by suppressing NF‐κB‐mediated inflammation.^15^ In addition, inhibition of IKKα and IKKβ increased recovery from fibrotic tissue in livers.^35^ Although most studies related to metabolic liver diseases have focused on canonical IKKα and IKKβ, Chiang and colleagues, however, reported that the non‐canonical IKKs, IKKε and TBK1 are more highly expressed than canonical activators in several tissues of obese animals with metabolic dysfunctions.^36^ Also, loss of TBK1 kinase activity protects high fat diet‐induced metabolic dysfunction in mice by interfering with inhibitory interaction between TBK1 and insulin receptor.^23^ Therefore, we hypothesize that the activation of TBK1 and IKKε may promote the liver fibrosis as well as metabolic liver diseases. Based on our results, increased protein levels of TBK1 and IKKε were observed in the livers of mice with hepatic fibrosis, which is consistent with increased these proteins in the livers of obese mice with metabolic disorder.^36^, ^37^ Notably, inhibition of TBK1 and IKKε by using amlexanox suppresses hepatic fibrogenesis and promoting the fibrosis resolution.

To demonstrate the mode of action of TBK1 and IKKε, we first ascertain the cellular source of these two kinases in livers. The results showed that various hepatic cell types including hepatocytes, KCs and HSCs expressed TBK1 and IKKε in physiologic or pathologic milieu. Especially, TBK1 and IKKε were predominantly expressed in KCs and HSCs in normal or fibrotic livers (Figure 1D). These findings further suggest a crucial role for TBK1 and IKKε in HSCs and KCs on the progression or resolution of hepatic fibrosis.

KCs, the mainly inflammatory regulators, were employed to clearly investigate the effect of TBK1 and IKKε to inflammation. In the present study, amlexanox treatment significantly decreased the inflammatory responses in LPS‐treated KCs via inhibiting the activity of NF‐κB. In addition to decrease of inflammatory responses, treatment of amlexanox significantly decreased the production of TGFβ in KCs as well as in hepatocytes, thereby contribute to decreasing the severity of liver fibrosis. As HSCs were the main cellular source of TBK1 and IKKε in fibrotic livers based on our results (Figure 1D), we further assumed that anti‐fibrotic effects of amlexanox might be caused by modulating TBK1 and IKKε of HSCs in fibrotic milieu. Consistent with this notion, our results showed that amlexanox could reverse the increased protein levels of TBK1 and IKKε in activated HSCs as well as in the fibrotic livers of mice.

Among various signalling pathways, the roles of NF‐κB and STAT3 in the pathogenesis of liver diseases have been extensively investigated.^33^ In addition to having beneficial effects on liver fibrosis by modulating NF‐κB, inhibition of STAT3 also exerts protective effects on fibrogenesis in livers.^38^, ^39^ Of note, recent finding has provided that activated NF‐κB and STAT3 could interact with each other leading to an increase of pro‐inflammatory cytokine secretion in HSCs.^40^ Although leptin or IL‐6 cytokine family stimulates STAT3 activation leading to increase of pro‐fibrogenic gene expression, a recent study has shown that crosstalk between STAT3 and TGFβ leads to pro‐fibrogenic responses via promoting activation and anti‐apoptosis of HSCs.^41^ In accordance with these findings, our results showed that anti‐fibrotic effects of amlexanox were, in part, mediated by reducing STAT3 activation in TGFβ‐treated LX‐2 cells and activated primary HSCs. Consistently, reduced STAT3 activity and anti‐fibrotic effects by amlexanox were abolished by treatment of STAT3 activator in activated HSCs. Conversely, treatment of STAT3 activation inhibitor further increased the anti‐fibrotic effects of amlexanox in activated HSCs. These results suggest that STAT3 was not fully inhibited by amlexanox, and anti‐fibrotic effects of amlexanox in activated HSCs were partially dependent on STAT3 activation. These results were further supported by recent finding, showing that TBK1 and IKKε play a crucial role in maintaining persistent activity of STAT3.^42^ Furthermore, recent studies have shown that NF‐κB protects activated HSCs against TNFα‐induced apoptosis,^43^, ^44^ and inhibition of NF‐κB led to the promotion of HSC apoptosis, resulting in reduced severity of liver fibrosis.^45^ Also, several studies have demonstrated that the apoptosis of activated HSCs is mechanistically implicated in the progression or resolution of liver fibrosis.^46^, ^47^ In line with these findings, treatment of amlexanox significantly increased the protein level of Bax (pro‐apoptosis protein), with a concomitant decrease of protein level of Bcl2 (anti‐apoptosis protein) in TGFβ‐treated LX‐2 cells and activated primary HSCs (Figure S6A,B). In line with these results, the ratio of Bax and Bcl2 was significantly increased by treatment of amlexanox in activated LX‐2 cells and primary HSCs (Figure S6C,D). Thus, inhibition of TBK1 and IKKε can promote the apoptosis of activated HSCs, which leads to a decrease of fibrogenic responses.

On the contrary, STAT3 in hepatocyte exerts hepatoprotective effects through enhancing proliferation of hepatocytes in livers with persistent damage and fibrosis.^48^, ^49^ Moreover, TBK1 and IKKε have protective functions against TNF‐induced cell death by receptor‐interacting serine/threonine‐protein kinase 1 phosphorylation.^50^ Therefore, cell type‐specific role of TBK1 and IKKε needs to be further determined the relationship between these two kinases and liver fibrosis.

Collectively, we have shown that the modulation of the TBK1/IKKε by amlexanox affects the activation of HSCs and KCs resulting in reduced fibro‐inflammatory responses. As more effective therapeutic strategies are urgently needed for treating hepatic fibrosis and cirrhosis, inhibition of TBK1 and IKKε by amlexanox may be a promising therapeutic strategy to cure fibrosis, which could allow for remodelling and regression of fibrosis.

## CONFLICT OF INTEREST

The authors declare that they have no conflict of interest.

## AUTHOR CONTRIBUTIONS

The study was conceived and designed by ZZ and J‐WK; ZZ, J‐WK. JQ performed the experiments and wrote the manuscript. JZ helped to collect the data. BK and CWL supervised the research and contributed to the critical review of the final manuscript.

## Data Availability

The data that support the findings of this study are available from the corresponding author upon reasonable request.
