# Supplementary figures and images for: Dual TBK1/IKKɛ inhibitor amlexanox attenuates the severity of hepatotoxin‐induced liver fibrosis and biliary fibrosis in mice
Source: J Cell Mol Med. 2019 Dec 10;24(2):1383–98. doi: 10.1111/jcmm.14817 (PMC6991653; doi:10.1111/jcmm.14817)

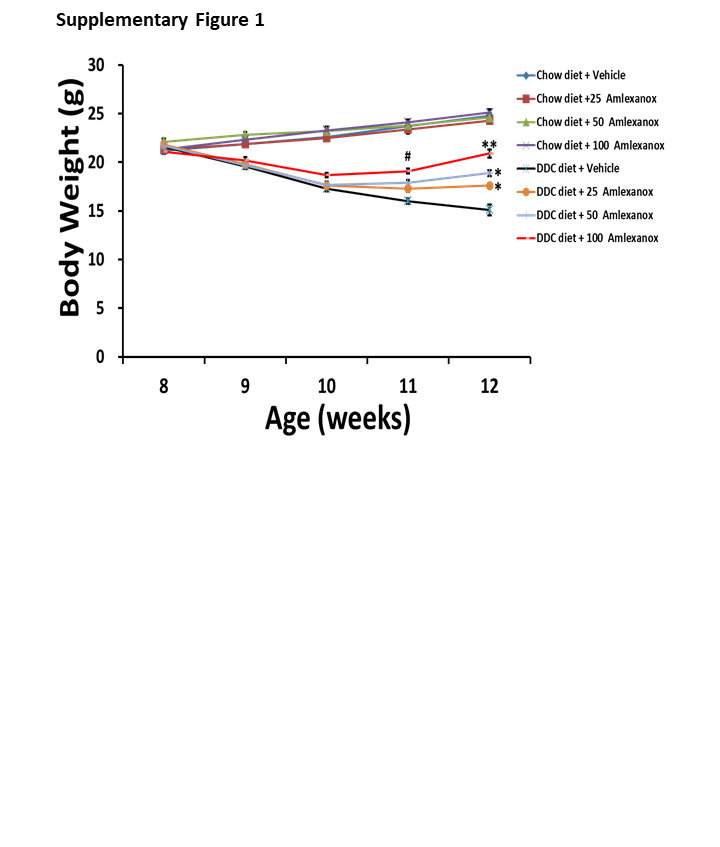

Supplement: Supplementary file 1 [file JCMM-24-1383-s001.TIF]

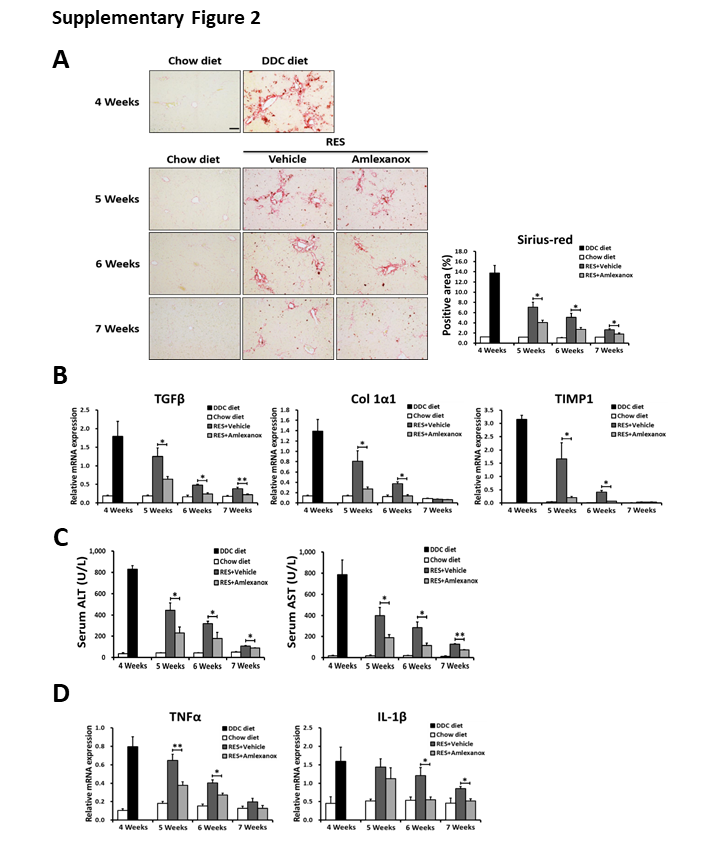

Supplement: Supplementary file 2 [file JCMM-24-1383-s002.TIF]

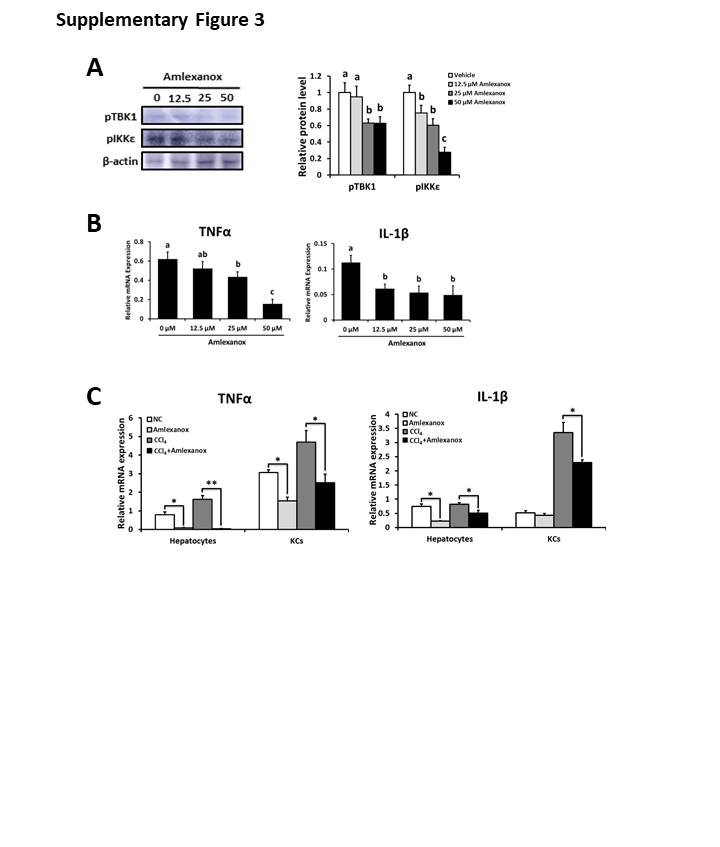

Supplement: Supplementary file 3 [file JCMM-24-1383-s003.TIF]

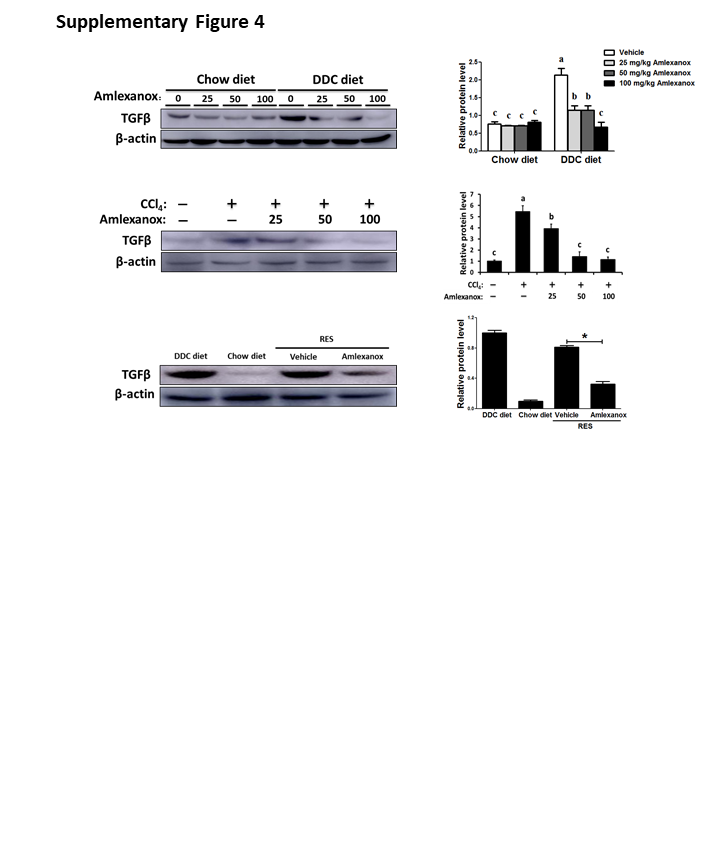

Supplement: Supplementary file 4 [file JCMM-24-1383-s004.TIF]

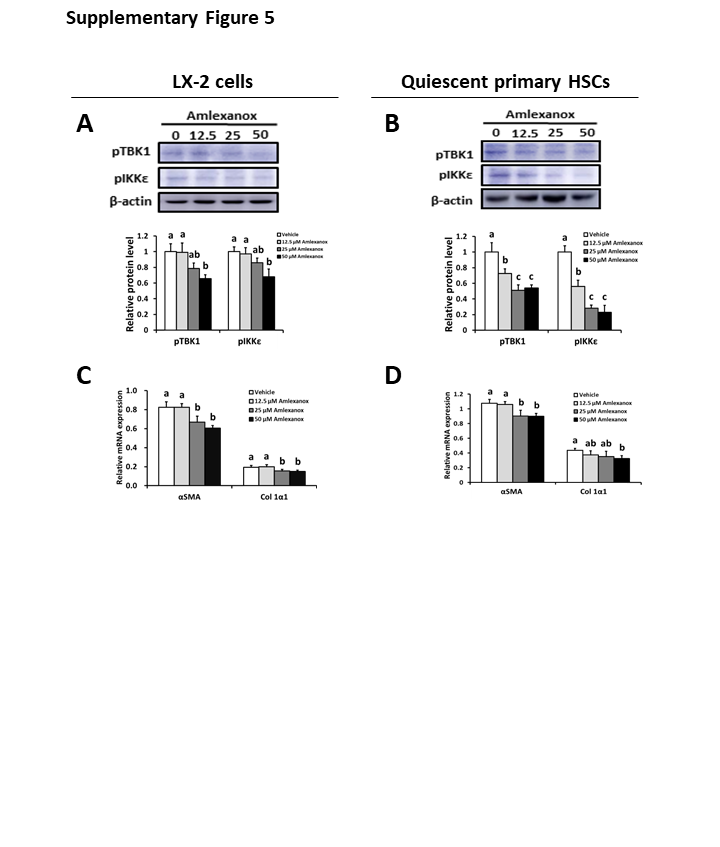

Supplement: Supplementary file 5 [file JCMM-24-1383-s005.TIF]

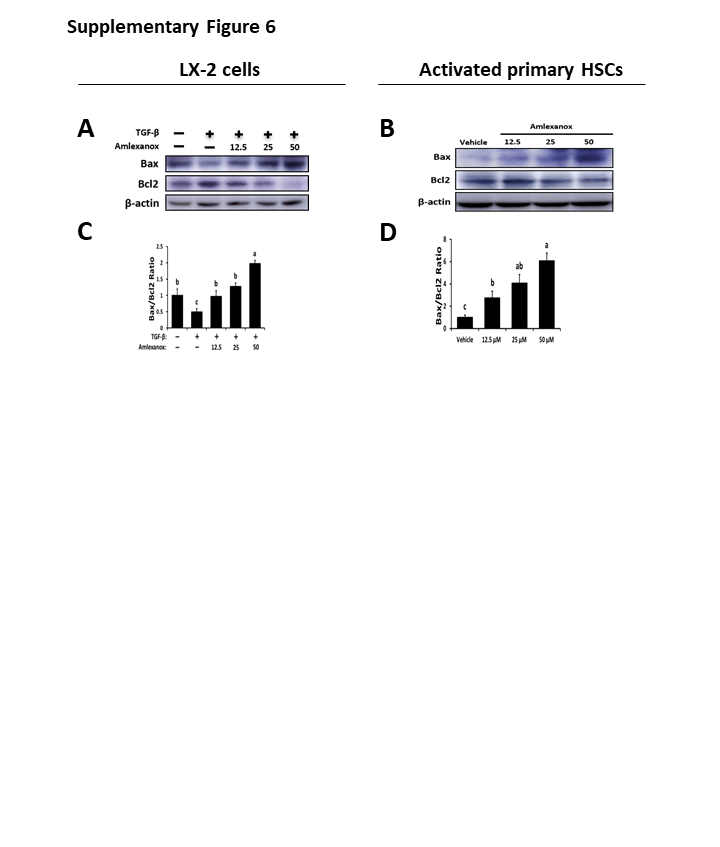

Supplement: Supplementary file 6 [file JCMM-24-1383-s006.TIF]
